# Dual Misuse of Insulin in an Adolescent With Type 1 Diabetes: A Case Report and Management Implications

**DOI:** 10.1155/crie/3651243

**Published:** 2026-02-16

**Authors:** Sara C. Pender, Mohamed Adil Shah Khoodoruth

**Affiliations:** ^1^ Department of Psychiatry, Western University Schulich School of Medicine and Dentistry, London, Ontario, Canada; ^2^ London Health Sciences Centre, London, Ontario, Canada, lhsc.on.ca; ^3^ Department of Child and Adolescent Psychiatry, Western University Schulich School of Medicine and Dentistry, London, Ontario, Canada

**Keywords:** case report, diabetic ketoacidosis (DKA), hybrid closed-loop insulin pump, self-harm, type 1 diabetes mellitus

## Abstract

**Introduction:**

In Canada, nearly one in five children present with diabetic ketoacidosis (DKA) at the time of their first diabetes diagnosis, a serious and potentially life‐threatening complication. Adolescence, marked by insulin resistance, fluctuating appetite, and increasing independence, presents unique challenges for diabetes management. Suicidality is more prevalent in adolescents with type 1 diabetes, with higher rates of ideation and attempts compared to peers without the condition. Insulin misuse, whether by omission or deliberate insulin overdose, is increasingly recognized as a maladaptive coping strategy linked to body image concerns, diabetes‐related distress, and psychiatric comorbidity, further compounding vulnerability.

**Case Report:**

A 15‐year‐old female with type 1 diabetes mellitus, diagnosed at age 11, was admitted to child and adolescent psychiatry following a suicide attempt by insulin overdose delivered via her Tandem X2 Control IQ insulin pump. She had a history of multiple suicide attempts, typically in the context of acute psychosocial stressors, and recently transitioned to foster care. Biochemistry revealed suboptimal glycemic control (HbA1c 8.5%) and mild metabolic acidosis. A multidisciplinary review led to targeted insulin adjustments and the introduction of a pump passcode lock, restricting unsupervised bolus dosing as a safeguarding measure against insulin overdose. Psychiatric management was optimized, and coordinated aftercare planning was arranged with child protection, endocrinology, and mental health services.

**Conclusion:**

This case illustrates insulin manipulation as a means of self‐harm in adolescence. The current admission involved a deliberate insulin overdose with mild DKA in the context of a history of insulin omission and more severe DKA episodes. Few published reports describe such dual misuse, underscoring the need to explicitly assess both behaviors in routine care. In this instance, reintroduction of pump therapy with passcode‐protected bolus delivery and tailored alarm configurations provided an additional safeguard, highlighting the potential for diabetes technology, when thoughtfully configured, to augment safety alongside psychiatric and psychosocial intervention.

## 1. Introduction

In Canada, nearly one in five children present in diabetic ketoacidosis (DKA) at the time of their first diabetes diagnosis, while national surveillance indicates that around 0.3% of those aged 1–19 live with diabetes, the vast majority (~95%) being type 1 diabetes (T1D) [1, 2]. Adolescence is among the most demanding stages of management, as physiological insulin resistance of puberty, fluctuating appetite, irregular sleep, and a growing drive for independence together render glycemic control particularly difficult. A recent meta‐analysis [3] reported a prevalence of suicidal ideation of 15.4% and suicide attempts of 3.5% in adolescents with T1D, higher than rates observed in peers without the condition (11.5% and 2.0%, respectively); suicide deaths ranged from 0.04% to 4.4% among adolescents with T1D. Given that suicide is the second leading cause of death among Canadian youth [4], the intersection of DKA and suicidality underscores a compounded vulnerability, where concurrent medical and psychiatric crises heighten the risk of adverse outcomes.

The withholding of insulin has long been recognized as a maladaptive coping strategy in some adolescents, often linked to body image dissatisfaction and the desire to control weight [5]. More recently, there has been increasing recognition that insulin may also be used in excess, not for metabolic benefit, but as a means of self‐harm [6].

For clinicians, the challenge lies in discerning intent and addressing risk on several fronts simultaneously. Recurrent DKA in particular is widely recognized as a marker of vulnerability, demanding more intensive, multidisciplinary intervention [2].

**Figure 1:**
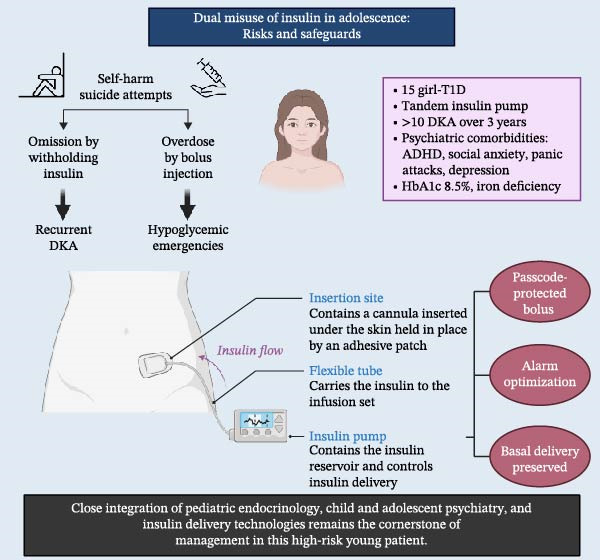
Dual misuse of insulin in adolescence: risks and safeguards.

In this report, we describe the case of a 15‐year‐old female with T1D whose clinical course has been marked by repeated and often severe episodes of DKA, resulting from both omission and deliberate overdose of insulin (Figure 1). Her trajectory illustrates the complex duality of insulin misuse in adolescence, involving maladaptive eating‐related behaviors, diabetes‐related distress, and suicidality. Beyond the description of her medical history, this case highlights the need for close collaboration between pediatric endocrinology and child and adolescent psychiatry. It also highlights the potential role of advanced diabetes technologies not only in achieving metabolic stabilization but also in enhancing patient safety. In this instance, a hybrid closed‐loop system was retained but configured with a bolus passcode, thereby preserving automated basal delivery while restricting manual bolus administration as a protective safeguard. To our knowledge, this may represent the first reported case of insulin misuse as a form of self‐harm in adolescents with T1D in Canada.

## 2. Case Presentation

The patient is a 15‐year‐old female diagnosed with T1D at age 11, an episode that was complicated by DKA, marking the beginning of a pattern of recurrent DKA episodes that later emerged in the context of insulin manipulation.

She is currently residing in a foster placement with several other children and an infant in care. She is due to commence grade 10 in the autumn. She was admitted to child and adolescent psychiatry following a suicidal attempt by deliberate insulin overdose delivered through her Tandem X2 insulin pump with Control IQ algorithm and Tru Steel infusion set, which was programed to deliver concentrated insulin lispro (U200). She also used a Dexcom G7 continuous glucose monitor.

**Figure 2:**
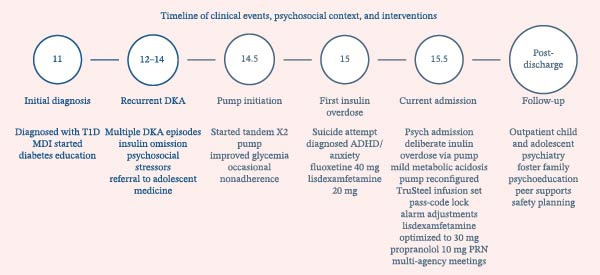
Timeline of clinical events, psychosocial context, and interventions.

She has a history of multiple suicidal attempts, either through withholding insulin, resulting in DKA, or by administering excessive insulin, typically in the context of acute psychosocial stressors. Her social circumstances are complex, as she began living with a new foster family last year. The patient had previously used the insulin pump without a passcode; however, glycemic outcomes during that period were suboptimal. She has established diagnoses of attention deficit hyperactivity disorder, managed with lisdexamfetamine 20 mg daily, and an unspecified anxiety/mood disorder, treated with fluoxetine 40 mg daily, and was followed by her family doctor and the adolescent medicine team. The electrocardiogram was normal. Biochemistry indicated suboptimal glycemic control (HbA1c 8.5%) with a mild high anion gap metabolic acidosis (anion gap 14 mmol/L, bicarbonate 21 mmol/L), alongside iron deficiency (reduced ferritin and serum iron) and isolated elevations of alkaline phosphatase and vitamin B12. The other bloodwork, such as complete blood count, thyroid function test, CRP, vitamin D, and folate, as well as ECG, was unremarkable. Figure 2 outlines the timeline of clinical events, psychosocial context, and interventions.

Insulin pump data were reviewed in collaboration with pediatric endocrinology (see Table 1). Targeted adjustments were implemented to optimize glycemic stability, and the pump was reissued with a passcode lock as a safeguarding measure against deliberate insulin overdose. During the current admission, the patient tolerated the passcode‐protected pump well, with no safety concerns observed on the inpatient unit or during leaves of absence.

**Table 1:** Summary of medical, technological, psychiatric, and safeguarding interventions implemented or considered during admission.

Intervention domain	On admission	Changes implemented/considered	Rationale/notes
Insulin pump parameters	Tandem X2 control IQ using insulin lispro U200	Adjusted basal rate (0.95→1.05 U/h mid‐day), ISF 1:4→1:3.5, added bolus passcode lock, maintained high‐glucose alarm	Optimize glycemic stability; mitigate risk of insulin overdose
Safety configuration	No bolus restriction; re‐alarm every 15 min	Passcode‐protected bolus, re‐alarm extended to 20 min, TruSteel infusion set	Reduce alarm fatigue; restrict unsupervised bolusing
Medication (psychiatric)	Fluoxetine 40 mg, lisdexamfetamine 20 mg	Increased lisdexamfetamine to 30 mg; added propranolol PRN for panic attacks	Optimize comorbid ADHD/anxiety management
Psychological support	Outpatient follow‐up only	Inpatient psychiatric care, Dialectical behavioral therapy, safety planning, and multi‐agency meetings (foster care, child protection, endocrinology)	Strengthen safeguarding and integrated risk management
Therapeutic supports	None structured	Referral to outpatient child and adolescent psychiatry; psychoeducation for foster family	Enhance continuity and support
Safety measures discussed but not applied	Long‐acting insulin (e.g., glargine) as safety back‐up	Not implemented due to existing pump compatibility, oversight strategy, and frequent follow‐ups with the diabetic nurse	Discussed with endocrinology but deemed unnecessary given pump reconfiguration and supervised setting
Community and psychosocial interventions	Limited supports pre‐admission	Community support and community therapy resources discussed at discharge planning, including involving assigned child protection worker	Enhance psychosocial resilience post‐discharge

Her psychiatric care was reviewed during the admission, and her lisdexamfetamine was optimized to 30 mg daily. On formal assessment, the Screen for Child Anxiety Related Emotional Disorders (SCARED) returned a total score of 53, with social anxiety and panic symptoms being the most pronounced. The Beck Depression Inventory–II (BDI‐II) score was 35, consistent with severe depressive symptoms.

A series of multi‐agency meetings was convened with her foster family, child protection services, endocrinology, and psychiatry to ensure a coordinated approach and to strengthen safeguarding measures. She remained an inpatient for 16 days. The patient has consistently expressed that the passcode‐protected pump felt safe and that her overall sense of well‐being improved following optimization of her SSRI and ADHD medications. Upon discharge, we prescribed propranolol as needed for panic attacks to help manage her performance‐related anxiety. We also requested that the adolescent medicine team arrange follow‐up with our outpatient child and adolescent mental health service, adding an additional layer of support and oversight to help mitigate ongoing risks.

## 3. Discussion

Intentional manipulation of insulin in adolescents with T1D has long been recognized, most commonly in adolescent girls. The behavior typically involves omission or restriction for weight‐related reasons, though in some instances, deliberate insulin overdosing is employed as a means of self‐harm. Whilst colloquially referred to as “diabulimia,” the phenomenon is more accurately understood as an expression of maladaptive eating‐related behaviors and diabetes‐related distress, often accompanied by comorbid psychiatric symptoms [7].

Epidemiological studies have consistently demonstrated that maladaptive eating‐related behaviors are highly prevalent in adolescents with T1D, affecting up to 60% of girls [8, 9]. Within this group, deliberate insulin omission or restriction is frequently reported with prevalence rates of ~11%–33%. Less frequently highlighted, but no less concerning, is the deliberate use of insulin in overdose for self‐harm. In a pediatric cohort studied by Schober et al. [10], 24% admitted to underdosing and 23% to overdosing, indicating that overuse may occur at rates comparable to omission. These findings underline the need for clinicians to assess explicitly both behaviors in a routine practice.

The present case represents an extreme manifestation along the spectrum of insulin misuse in adolescents. This 15‐year‐old female engaged in both omission and deliberate overdose of insulin, each with self‐harm intent, resulting in more than 10 admissions with DKA in 3 years, several of which involved metabolic derangement. While the literature contains few reports of adolescents exhibiting both insulin omission and deliberate insulin overdose, this case uniquely illustrates the dual pathways through which insulin misuse may present. It highlights the complex interplay between maladaptive eating‐related behaviors and suicidality and underscores the grave risks that arise when insulin functions both as a life‐sustaining therapy and a potential means of self‐injury.

To our knowledge, this may be the first case report from Canada to specifically highlight this dual pattern of insulin misuse in adolescence. Although a 2020 study from Quebec by Robinson et al. [11] reported that the risks of psychiatric disorders and suicide attempts were substantially higher among adolescents and emerging adults with diabetes mellitus compared to their peers without diabetes, that study did not describe the methods or contexts of the suicide attempts, making the present case a unique contribution to the literature. Additionally, a recent systematic review and meta‐analysis [3] on suicidality in individuals with diabetes did not appear to include any similar case reports, referencing only the Quebec study, which further underscores the novelty of our findings.

Management in such cases demands multidisciplinary collaboration. Pediatric endocrinology and diabetes nursing teams are often the first to recognize aberrant insulin patterns, but psychiatric assessment is essential to ascertain intent and address underlying drivers. The therapeutic plan in this case also highlighted the limitations of multiple daily injections (MDI). On MDI, the patient retained unrestricted access to both basal and bolus insulin, allowing her to administer excessive doses or omit entirely with little opportunity for oversight. Transitioning back to pump therapy offered not only metabolic advantages but also new avenues for safeguarding. The passcode‐protected bolus function restricted unsupervised access to large correction doses, while basal profiles and insulin sensitivity factors could be more finely titrated to stabilize glycemia. Reconfigured alarm thresholds and re‐alarm intervals reduced hypoglycemic risk while addressing alarm fatigue. While these safeguards limit unsupervised access to high‐risk correction doses and reduce the likelihood of hypoglycemia, the potential for harm remains; patients retain the ability to suspend or disconnect insulin delivery entirely, leaving the risk of DKA unmitigated in certain scenarios.

There is increasing evidence that advanced diabetes technologies, particularly hybrid closed‐loop systems, provide more than metabolic benefit. They may also reduce psychological burden, support family supervision, and offer clinicians additional tools for safeguarding vulnerable adolescents [3, 12, 13]. In more vulnerable patients such as this, thoughtful configuration of pump functions, alarms, and safety locks may help to bridge the gap between medical stabilization and the longer‐term work of psychological intervention.

This case, therefore, underscores both the severity of intentional insulin misuse and the potential for technology to mitigate, though not eliminate, risk. The contrast between MDI and pump therapy in this instance demonstrates how technology, when deliberately adapted to a clinical context, can serve as an additional safeguard alongside psychiatric and psychosocial care. Although this report highlights a unique presentation of dual insulin misuse, it remains a single case. Longitudinal outcomes are not yet known, and the generalizability is limited. Moreover, while pump safety features reduced the likelihood of insulin overdose, they do not prevent deliberate disconnection or suspension of delivery. This reinforces the need for close integration of pediatric endocrinology, child and adolescent psychiatry, and the judicious use of advanced diabetes technologies, which remain the cornerstone of management in this challenging population.

## 4. Conclusion

This case illustrates the severe risks associated with insulin manipulation in adolescence, where both omission and deliberate insulin overdose were employed with self‐harm intent, resulting in recurrent and life‐threatening DKA. Few published reports describe such dual misuse, underscoring the importance of explicit inquiry into both behaviors in routine clinical practice. The management of such young people requires close integration of pediatric endocrinology, diabetes nursing, and child and adolescent mental health services. In this instance, the use of insulin pump therapy, configured with safety features such as passcode‐protected bolus delivery and tailored alarm settings, provided an additional layer of safeguarding and metabolic stability. While technology alone cannot resolve the underlying psychosocial drivers, its deliberate use alongside psychiatric care may offer valuable protection during periods of heightened risk.

## Author Contributions

**Sara C. Pender**: writing – original draft preparation, investigation, visualization, writing – reviewing and editing, project administration. **Mohamed Adil Shah Khoodoruth**: conceptualization, visualization, writing – reviewing and editing, supervision, funding acquisition.

## Funding

This study did not receive specific project funding; however, Dr. Mohamed Adil Shah Khoodoruth is supported by research funding from Western University, Schulich School of Medicine and Dentistry.

## Consent

The authors confirm that the written informed consent for the publication of the case details was obtained from the patient.

## Conflicts of Interest

The authors declare no conflicts of interest.

## Data Availability

Data sharing is not applicable to this article, as no datasets were generated or analyzed during the current study.

## References

[1] Public Health Agency of Canada , Twenty Years of Diabetes Surveillance Using the Canadian Chronic Disease Surveillance System (CCDSS), 1995 to 2015, Health Promotion and Chronic Disease Prevention in Canada. (2019) 39, no. 11, 306–309, 10.24095/hpcdp.39.11.03. 31729313 PMC6876649

[2] Wherrett D. K. , Ho J. , Huot C. , Legault L. , Nakhla M. , and Rosolowsky E. , Type 1 Diabetes in Children and Adolescents, Canadian Journal of Diabetes. (2018) 42, no. Suppl 1, S234–S246, 10.1016/j.jcjd.2017.10.036, 2-s2.0-85044754701. 29650103

[3] Renaud-Charest O. , Stoljar Gold A. , Mok E. , Kichler J. , Nakhla M. , and Li P. , Suicidal Ideation, Suicide Attempts, and Suicide Deaths in Adolescents and Young Adults With Type 1 Diabetes: A Systematic Review and Meta-Analysis, Diabetes Care. (2024) 47, no. 7, 1227–1237, 10.2337/dc24-0411. 38900947

[4] Statistics Canada , Leading Causes of Death, Total Population, by Age Group, 2025, Government of Canada, https://www150.statcan.gc.ca/t1/tbl1/en/tv.action?pid=1310039401.

[5] Garrido-Bueno M. , Núñez-Sánchez M. , García-Lozano M. S. , Fagundo-Rivera J. , Romero-Alvero A. , and Fernández-León P. , Effects of Body Image and Self-Concept on the Management of Type 1 Diabetes Mellitus in Adolescents and Young Adults: A Systematic Review, Healthcare. (2025) 13, no. 12, 10.3390/healthcare13121425, 1425. 40565452 PMC12193616

[6] Russell-Jones D. and Khan R. , Insulin-Associated Weight Gain in Diabetes–Causes, Effects and Coping Strategies, Diabetes, Obesity and Metabolism. (2007) 9, no. 6, 799–812, 10.1111/j.1463-1326.2006.00686.x, 2-s2.0-35048841093. 17924864

[7] Colton P. , Olmsted M. , Daneman D. , Rydall A. , and Rodin G. , Disturbed Eating Behavior and Eating Disorders in Preteen and Early Teenage Girls With Type 1 Diabetes: A Case-Controlled Study, Diabetes Care. (2004) 27, no. 7, 1654–1659, 10.2337/diacare.27.7.1654, 2-s2.0-3042834431. 15220242

[8] Goebel-Fabbri A. E. , Disturbed Eating Behaviors and Eating Disorders in Type 1 Diabetes: Clinical Significance and Treatment Recommendations, Current Diabetes Reports. (2009) 9, no. 2, 133–139, 10.1007/s11892-009-0023-8, 2-s2.0-63349083562. 19323958

[9] Young V. , Eiser C. , and Johnson B. , et al.Eating Problems in Adolescents With Type 1 Diabetes: A Systematic Review With Meta-Analysis, Diabetic Medicine. (2013) 30, no. 2, 189–198, 10.1111/j.1464-5491.2012.03771.x, 2-s2.0-84872700095. 22913589

[10] Schober E. , Wagner G. , and Berger G. , et al.Prevalence of Intentional Under- and Overdosing of Insulin in Children and Adolescents With Type 1 Diabetes, Pediatric Diabetes. (2011) 12, no. 7, 627–631, 10.1111/j.1399-5448.2011.00759.x, 2-s2.0-80055096728. 21435136

[11] Robinson M.-E. , Simard M. , Larocque I. , Shah J. , Nakhla M. , and Rahme E. , Risk of Psychiatric Disorders and Suicide Attempts in Emerging Adults With Diabetes, Diabetes Care. (2020) 43, no. 2, 484–486, 10.2337/dc19-1487. 31843949

[12] Urakami T. , The Advanced Diabetes Technologies for Reduction of the Frequency of Hypoglycemia and Minimizing the Occurrence of Severe Hypoglycemia in Children and Adolescents With Type 1 Diabetes, Journal of Clinical Medicine. (2023) 12, no. 3, 10.3390/jcm12030781. PMC9917934 36769430

[13] Olid-Cárdenas M. J. , Lendínez-Jurado A. , and Monroy-Rodríguez G. , et al.Real-World Use of Hybrid Closed-Loop Systems During Diabetes Camp: A Preliminary Study for Secure Configuration Strategies in Children and Adolescents, Nutrients. (2024) 16, no. 14, 10.3390/nu16142210, 2210. 39064653 PMC11279836

